# Two novel red-FRET ERK Biosensors in the 670-720 nm range

**DOI:** 10.1186/s13036-025-00541-9

**Published:** 2025-11-13

**Authors:** Nicholaus L. DeCuzzi, Jason Y. Hu, Florene Xu, Brayant Rodriguez, Michael Pargett, John G. Albeck

**Affiliations:** Department of Molecular and Cellular Biology, University of California Davis, CA Davis, USA

## Abstract

Cell fate decisions are regulated by intricate signaling networks, with Extracellular signal-Regulated Kinase (ERK) being a central node in the control of cell proliferation and differentiation. ERK typically cooperates with a network of other regulators, making it necessary to study multiple signaling pathways simultaneously at the single-cell level. Many existing fluorescent biosensors for ERK and other pathways have significant spectral overlap, limiting their utility for multiplexing. To address this limitation, we developed two novel red-FRET ERK biosensors, REKAR67 and REKAR76, which operate in the 670–720 nm range using the fluorescent proteins miRFP670nano3 and miRFP720. REKAR67 and REKAR76 differ in fluorophore position, which impacts biosensor characteristics; REKAR67 displayed a higher dynamic range but greater signal variance than REKAR76. In both polyclonal and clonal populations, REKAR67- or REKAR76-expressing cells displayed similar Signal-to-Noise ratios (*SNR*). Overall, the red-FRET ERK biosensors were highly consistent with existing CFP/YFP biosensors in reporting ERK activity. Both REKAR biosensors expand the available tools for measuring single-cell ERK activity by being spectrally compatible with other CFP/YFP FRET and cpGFP -based biosensors, allowing for multiplexed imaging.

**Graphical Abstract:**

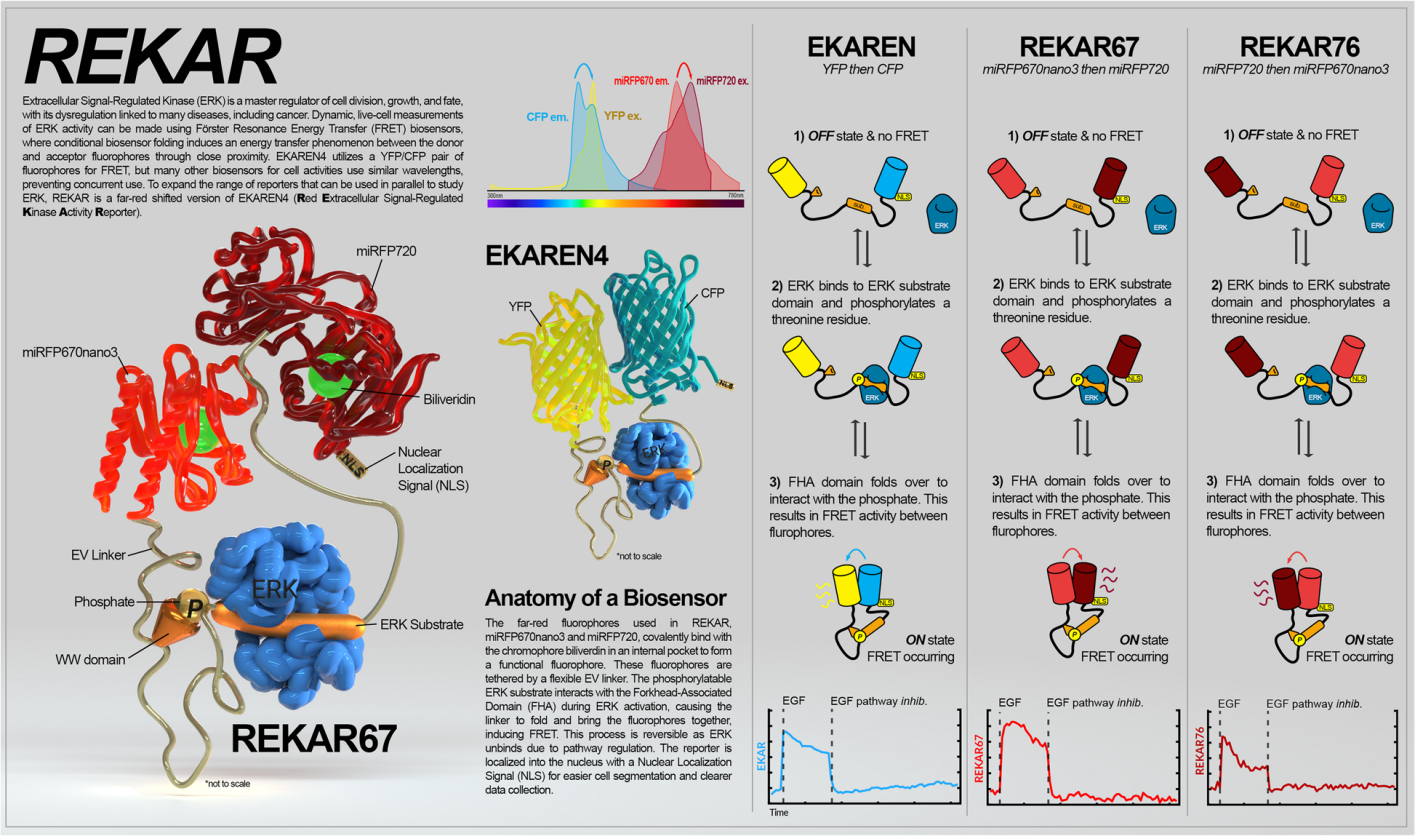

**Supplementary Information:**

The online version contains supplementary material available at 10.1186/s13036-025-00541-9.

## Introduction

Extracellular signal-Regulated Kinases 1 and 2 (ERK) are terminal kinases of the Mitogen Activated Protein Kinase (MAPK) signaling pathway and regulate cell fates including proliferation, growth, and apoptosis [1, 2]. These cell fate decisions are determined by the temporal activity patterns of ERK [3, 4], motivating the need to quantify ERK activity at the single-cell level. Fluorescent biosensors have revealed significant insight into the spatiotemporal signaling activity of ERK, providing real-time activity readouts with little perturbation to normal kinase function [5]. For example, ERK activity often occurs in radiating waves originating at discrete points, both in vivo [6] and in various cell culture models [7–10]. These waves have functional significance in maintaining homeostasis of epithelial layers [9]. Furthermore, oncogenic mutations in RAS or Epidermal Growth Factor (EGF) Receptor (EGFR) modulate the forms of ERK kinetics [11, 12], impacting the expression of downstream genes [13]. A key area of interest is to understand how these dynamic ERK activity patterns integrate with other signaling pathways. However, a significant barrier for multi-biosensor experiments is the spectral incompatibility and substrate specificity of many currently available biosensors. 

One widely used design for fluorescent biosensors employs Förster Resonance Energy Transfer (FRET) between two coupled fluorophores, where the excitation energy of one (FRET donor) is transferred to and subsequently emitted by the second fluorophore (FRET acceptor). ERK Kinase Activity Reporters (EKARs) are a class of FRET biosensors that typically use cyan and yellow fluorescent proteins (CFP and YFP) as a FRET pair to quantify ERK activity in living cells. For example, the EKAR-EV biosensor contains the CFP variant ECFP tethered to the YFP variant YPet via a flexible linker [14]. Within this linker region, there is an ERK substrate recognition domain containing a phospho-acceptor threonine residue, and a WW phospho-amino acid binding domain. When the threonine residue within the ERK substrate domain is phosphorylated, it is recognized and bound by the WW domain, forming an intramolecular interaction that brings the two fluorophores into close proximity and increases FRET efficiency, which can be measured by ratiometric imaging.

There have been multiple improvements to EKARs since their initial inception [15], including changes to the linker length [16], usage of brighter and more pH-stable CFP and YFP variants [14], and changes in FP position. Recent iterations, EKAREN4 and EKAREN5, addressed the non-specific phosphorylation of the biosensor by CDK1, which causes non-ERK-specific signals in G2/M phases of the cell cycle [17]. Two amino acid substitutions in the substrate domain were sufficient to nearly eliminate the CDK-mediated response of the biosensor. By making changes in linker length, EKAREN4 and EKAREN5 were further modified to have different dynamic ranges relative to ERK activity. Consequently, EKAREN4 has a larger range overall and higher saturation point, while EKAREN5 has a greater degree of sensitivity and is best for detecting low levels of ERK activity [17].

While CFP/YFP FRET-based ERK biosensors offer valuable insights into ERK activity, their spectral overlap with many other biosensors limits the ability to simultaneously monitor multiple signaling pathways, which is crucial for understanding complex cell fate decisions. For example, measuring ERK and Protein Kinase B (AKT) activity together can provide insights into their combined roles in promoting cell proliferation [9, 18–20]. However, one of the best-performing tools available for AKT activity detection, the cpGFP-based AKT biosensor ExRai-AKTAR2 [21], cannot be easily multiplexed with EKAR biosensors because it has overlapping spectral properties with CFP and YFP. The development of spectrally distinct fluorescent biosensors would alleviate this issue and allow for multiple signaling pathways to be measured with subcellular resolution in the same cell. However, the possibilities for red/far-red FRET versions of CFP/YFP FRET biosensors remain largely unexplored. Furthermore, while the position of FPs (N-terminal vs. C-terminal) has varied across biosensor versions [16, 22], the influence of this parameter on performance has not been systematically tested.

Here, we addressed the wavelength limitation in EKAR biosensors by developing two red-shifted versions of EKAREN4, designated as REKAR67 and REKAR76. These new constructs operate in the 670–720 nm range, using the fluorophores miRFP670nano3 and miRFP720 as FRET donor and acceptor, respectively. The two versions of REKAR were created by alternating the order of the two red FPs and were used to determine if FP position impacts biosensor characteristics, including dynamic range, noise variance, and Signal to Noise Ratio (*SNR*). Of the two biosensors, we found that REKAR67 has a better dynamic range, while REKAR76 has reduced signal variance. By comparing each REKAR variant directly to EKAREN4 in dual biosensor-expressing cells, we found that both REKAR versions are comparable to EKAREN4 in response to Epidermal Growth Factor (EGF) stimulation and EGFR and MEK inhibition.

## Materials and methods

Key resources table.

**Table:** 

Reagent or resource	Source	Identifier
**Chemicals, Peptides, and Recombinant Proteins**		
Collagen I, Rat tail	Gibco	Cat#A1048301
Epidermal Growth Factor (EGF)	Peprotech	Cat#AF-100–15
PD-0325901	Selleck Chemicals	Cat#S1036
Gefitinib (ZD1839)	Selleck Chemicals	Cat#S1025
FuGENE® HD Transfection Reagent	FuGENE	HD-1000
Gibson Assembly Master Mix	New England BioLabs	M5510A
**Experimental Models: Cell Lines**		
MCF-10A, Clone 5e	Joan Brugge, HarvardMedical School	RRID: CVCL_0598
MCF-10A-EKAREN4	This report	N/A
MCF-10A-EKAREN4-REKAR67	This report	N/A
MCF-10A-EKAREN4-REKAR67-T/A	This report	N/A
MCF-10A-EKAREN4-REKAR76	This report	N/A
MCF-10A-EKAREN4-REKAR76-T/A	This report	N/A
MCF-10A-REKAR67	This report	N/A
MCF-10A-REKAR67-T/A	This report	N/A
MCF-10A-REKAR76	This report	N/A
MCF-10A-REKAR76-T/A	This report	N/A
**Recombinant DNA**		
pLV x EF1a_EKAREN4-ires-puro	Ponsioen et al. 2021	Addgene#167828
pLenti-REKAR67-nls-puro	This report	Addgene#223261
pLenti-REKAR67-TA-nls-puro	This report	Addgene#225706
pLenti-REKAR76-nls-puro	This report	Addgene#223262
pLenti-REKAR76-TA-nls-puro	This report	Addgene#225705
**Software and Algorithms**		
NIS-Elements AR ver. 4.20	Nikon	RRID: SCR_014329
MATLAB	Mathworks	RRID: SCR_001622
Bio-Formats	OME	RRID: SCR_000450
Affinity Designer 2	Serif	N/A
WOMP3D	Womp	N/A
**Other**		
Glass bottom plates, #1.5H high performance cover glass	Cellvis	Cat#P96-1.5H-N, P06-1.5H-N

### Cell culture and media

Human mammary epithelial cells, MCF-10A clone 5E, were cultured in MCF-10A Growth Medium (see Tables 1, 2, 3 for media composition) as described in [23]. Low passage stocks from the parental MCF-10A-5E clone were used to create REKAR67, REKAR67-T/A, REKAR76, and REKAR76-T/A containing cell lines. An MCF-10A clone that expresses EKAREN4 was used to create EKAREN4-REKAR67, EKAREN4-REKAR67-T/A, EKAREN4-REKAR76, and EKAREN4-REKAR76-T/A cell lines. All cell lines were passaged before reaching 85% confluency.

**Table 1:** MCF-10A Growth Medium (GM)

Reagent	Supplier	Item #	Stock Concentrations	Final Concentrations
DMEM/F12	Gibco	11,320,033	1X	1X
Horse Serum	Gibco	26,050,088	100% v/v	5% v/v
Cholera Toxin	Sigma	C8052	1 mg/mL	100 ng/mL
EGF	Peprotech	AF-100–15	100 μg/mL	5 ng/mL
Hydrocortisone	Sigma	H0888	1 mg/mL	0.5 μg/mL
Insulin	Sigma	I9278	10 mg/mL	10 μg/mL
Biliverdin*	Sigma	30,891	32 mM	25 μM

^*^Only supplemented into growth medium 24–48 h prior to use for an imaging experiment

**Table 2:** MCF-10A Imaging Medium (IM)

Reagent	Supplier	Item #	Stock concentrations	Final concentrations
Imaging base- DMEM/F12*	UC Davis Veterinary Medicine Biological Media Services		1X	1X
Bovine Serum Albumin	Sigma	A7906	1X	0.1% w/v
Glucose	Fisher		1.74 M	17.4 mM
Pyruvate	Gibco	11,360,070	100 mM	1 mM
Hydrocortisone	Sigma	H0888	1 mg/mL (2.76 mM)	0.5 ug/mL (1.38 μM)
Cholera Toxin	Sigma	C8052	1 mg/mL	100 ng/mL

^*^ Custom F12/DMEM similar to GIBCO 11320033 lacking Glucose, Glutamine, Pyruvate, Folic Acid, Riboflavin, and Phenol Red

**Table 3:** MCF-10A Resuspension Medium

Reagent	Supplier	Item #	Stock concentrations	Final concentrations
DMEM/F12	Gibco	11,320,033	1X	1X
Horse Serum	Gibco	26,050,088	1X	10% v/v

### Biosensor construction

REKAR67 and REKAR76 plasmids were assembled by replacing the YFP and CFP fluorophores of pLJM1-EKAREN4 with miRFP670nano3 and miRFP720 from synthesized fragments via Gibson assembly (NEB M5510A). REKAR67 is defined by the N-terminal location of miRFP670nano3, miRFP720. REKAR76 is characterized by the N-terminal location of miRFP720 followed by miRFP670nano3. The negative controls, REKAR67-T/A and REKAR76-T/A, were generated with a T498A mutation, preventing phospho-recognition of the substrate domain.

### Biosensor delivery and cell line development

The biosensors REKAR67 or REKAR76 were transduced into MCF-10A-5E cells, or into MCF-10A-5E cells with a previous clonal integration of EKAREN4, via lentiviral transduction with pLenti-REKAR viral particles [24]. Lentiviral particles were synthesized by transfecting HEK 293 T cells with the Fugene HD transfection reagent (SKU: HD-1000). Lentivirus transduction was carried out in the presence of 8 μg/mL polybrene, with centrifugation at 100 × g for 30 min. Flow cytometry sorting was used to isolate biosensor positive cells with a Beckman Coulter MoFlo Astrios EQ cell sorter (UC Davis Flow Cytometry Shared Resource). Polyclonal populations of REKAR- positive cells were isolated by bulk sorting. Clonal REKAR lines were isolated from the polyclonal population by single-cell sorting into individual wells of 96-well plates. Each well was imaged in brightfield, RFP670, and FRET720 channels to confirm that clonal populations expressed the REKAR biosensor homogenously and were not contaminated by other cell populations. Out of the clonal REKAR populations isolated (3 clones per REKAR isoform), 2 clones of each REKAR biosensor were selected for further analysis and use in this manuscript.

### Live-cell microscopy experiments

Live-cell imaging experiments were conducted following previously described protocols [25–27]. MCF-10A cells were plated on 96-well imaging plates (Cellvis #:P96-1.5H-N) at least 48 h prior to experiments. Cells were seeded in MCF-10A Growth Medium supplemented with 25 μM biliverdin, then transferred to MCF-10A Imaging Medium at least 16 h prior to imaging (see Media Composition tables). During live-cell imaging experiments, cells were maintained at 37 °C with 5% CO_2_. Images of cells were taken every 6 min using a Teledyne Photometrics Kinetix sCMOS camera and a Nikon (Tokyo, Japan) 20X/0.75 NA Plan Apo objective on a Nikon Eclipse Ti2 inverted microscope, equipped with a Lumencor SPECTRA III light engine. Experiments were conducted using the following fluorescence filter sets: CFP (#49,001, Chroma), YFP (#49,003, Chroma), RFP670 (custom T647lpxr – ET667/30 m, Chroma, with the 635/22 nm SPECTRA III excitation band) and FRET720 (custom T647lpxr – FF01-730/39–32, Chroma and Semrock, with the 635/22 nm SPECTRA III excitation band). EKAREN4 measurements were made using CFP and YFP filters, and REKAR measurements were made using miRFP670 and FRET720 filters.

### Biosensor quantification

The EKAREN4 biosensor was quantified as described in [25]. Briefly, the response of the EKAREN4 biosensor is represented by the value $E{f}_{A}$, the product of $E$, the FRET efficiency of the fluorophore pair in the associated configuration, and ${f}_{A}$, the fraction of fluorophore pairs in that associated configuration. This value is computed as: $E{f}_{A} = 1- \left(\frac{CFP Intensity}{YFP Intensity} / {R}_{P}\right)$, where ${R}_{P}$ is the ratio of total channel gain in the Donor (CFP) channel divided by that in the Acceptor (YFP) channel. Each gain is computed as the spectral product of the contributing imaging system parameters: the relative excitation intensity, exposure time, molar extinction coefficient, quantum yield, light source spectrum, filter transmissivities, and fluorophore absorption and emission spectra.

The response of the REKAR biosensor was determined from the same underlying model. However, given the spectra of the miRFP670nano3 and miRFP720 fluorophores, it is not feasible to measure their contributions without significant spectral overlap. Accordingly, a more general form of the FRET measurement model was necessary to account for spectral crosstalk terms. In our imaging system, and many with laser-based light sources, there is no efficient option for preferentially exciting miRFP720 to measure the acceptor intensity. Instead, we measure the Donor fluorescence (via the RFP670 filter set), and the FRET fluorescence (via the FRET720 filter set). Working from the imaging system model presented in the “FRET reporter measurement and correction” section of the appendix to reference [12], we reformulate (from Eq. 3 onward) to model the intensity ratio of the FRET720 imaging channel to the RFP670 channel as:

1 $$\frac{I_FRET720}{I_RFP670}=\frac{P_Ft_F\int(C^DX_F^DM_F^D(1-Ef_A)+C^DX_F^DM_F^AEf_A+C^AX_F^AM_F^A)d\lambda}{P_Dt_D\int(C^DX_D^DM_D^D(1-Ef_A)+C^DX_D^DM_D^AEf_A+C^AX_D^AM_D^A)d\lambda}\downarrow\\$$

Herein, $P$ denotes the relative power delivered, $t$ the exposure time, $C$ concentration of a fluorophore, $X$ an excitation spectrum (the spectral product of the light source spectrum, the excitation filter, and the fluorophore absorption spectrum), and $M$ an emission spectrum (product of the fluorophore quantum yield, emission spectrum and the emission filter transmissivity). In superscripts, D refers to the Donor fluorophore (miRFP670nano3), and A the acceptor (miRFP720). In subscripts, D refers to the Donor imaging channel (via the RFP670 filter set), and F the FRET imaging channel (via the FRET720 filter set).

In this case, the spectral cross-talk term for the Donor emission, ${M}_{D}^{A}$ (the fraction of light emitted by the Acceptor that is collected, relative to that collected from the Donor), is estimated to be on the order of 0.1 or less, confirmed by the absence of signal in the RFP670 channel from isolated miRFP720 molecules (data not shown). This allows simplification of the denominator of the spectral ratio model by neglecting this term:

2 $$\frac{{I}_{FRET720}}{{I}_{RFP670}}=\frac{{P}_{F}{t}_{F}\int \left({C}^{D}{X}_{F}^{D}{M}_{F}^{D}\left(1-E{f}_{A}\right) +{ C}^{D}{X}_{F}^{D}{M}_{F}^{A}E{f}_{A} + {C}^{A}{X}_{F}^{A}{M}_{F}^{A}\right)d\lambda }{{P}_{D}{t}_{D}\int {C}^{D}{X}_{D}^{D}{M}_{D}^{D}\left(1-E{f}_{A}\right) d\lambda }$$

This model may then be rearranged, employing several substitutions for convenience. Define the normalized intensity ratio (NIR), and spectral ratios for cross-talk (XT), channel, and FRET:

3 $$NIR=\left(\frac{I_{FRET720}}{I_{REP670}}\right)/\left(\frac{P_Ft_F}{P_Dt_D}\right),\;R_{XT}=\frac{\int X_F^DM_F^Dd\lambda}{\int X_D^DM_D^Dd\lambda},\;R_{Chan}=\frac{\int X_F^AM_F^Ad\lambda}{\int X_D^DM_D^Dd\lambda},\;R_{FRET}=\frac{\int X_F^DM_F^Ad\lambda}{\int X_D^DM_D^Dd\lambda}$$

Rearranging to collect $E{f}_{A}$ uniquely on the left-hand side, the final model may then be written as:

4 $$E{f}_{A} = \frac{NIR - {R}_{XT} - \frac{{ C}^{A}}{{ C}^{D}}{R}_{Chan}}{NIR -{ R}_{XT} + {R}_{FRET}}$$

Each of the three spectral “ratios” may be computed via calibrations of the imaging system. Because the crosstalk ratio (${R}_{XT}$) is largely composed of effects on the edges/tails of spectra, it may be necessary to make a direct calibration measurement of the interaction. Some variance exists in in the spectra provided by light source and filter manufacturers, as well as those reported for the fluorophores. At the edges/tails, where spectral power or transmissivity is changing rapidly with wavelength, these minor errors in different spectra may stack to yield significant errors in the computed spectral products and ratios. By imaging the miRFP670nano3 fluorophore alone via both the RFP670 and FRET720 filter sets, the crosstalk ratio was directly measured, with a value of 0.578 on our hardware (data not shown), which in this case is less than 5% greater than the computed value of 0.551.

### Single-cell data processing

Single-cell values for the fractions of active EKAREN4 and/or REKAR biosensors were quantified, tracked over time, and filtered to omit data tracks that did not last > 85% of the movie length or that showed signals outside of expected ranges (EKAREN4 = 0.3—0.65 & all REKAR variants = 0.15—0.4). For reporter comparisons, data were normalized by the average scaling factor between REKAR and EKAREN4 signals across all cells for 3 experimental replicates with at least 3 technical replicates each. This scaling was performed by: 1) smoothing individual cell time series with a 3-point moving average, 2) subtracting the minimum smoothed value from each time series, and 3) calculating the average REKAR-to-EKAR scaling factor as the average of the ratio of the REKAR and EKAREN4 signal in each cell. The REKAR-to-EKAR scaling factors across all cells were subsequently used to scale all plotted raw REKAR data to the EKAREN4 data (REKAR67 = 1.5679; REKAR76 = 3.0042). When plotting normalized data for figures, after following steps 1 and 2 of the scaling outlined above, single cell data were multiplied by the REKAR-to-EKAREN4 scaling factor (EKAREN4 data was multiplied by 1), then the signal was multiplied by a normalizing value to scale the signal to range from 0–1 (REKAR67 = 5; REKAR76 = 3; EKAREN4 = 4). Data normalization was performed on the EKAREN4, REKAR67, and REKAR76 biosensor signals of all cells as indicated. Code for this scaling is available in the REKAR GitHub.

### Determination of biosensor dynamic range, variance and signal to noise ratio (SNR), and statistical analysis

To compare the REKAR and EKAREN4 biosensors we measured the dynamic range, variance, and signal to noise ratio at the single cell level. Code for this analysis is available in the REKAR GitHub. Briefly, biosensor expressing cells were sequentially exposed to an ERK activator (to obtain maximum biosensor signal) and EGFR & MEK inhibitors (to obtain minimal biosensor signal). At the single-cell level, biosensor dynamic range was determined by subtracting the cell’s minimum biosensor signal from the maximum signal measured. Biosensor variance was determined by taking the variance of the signal across 3 h following inhibitor treatment starting 2 h after treatment (hours 2 to 5 following inhibitor treatment. Biosensor *SNR* was determined by taking the single-cell dynamic range and dividing it by its own variance (N ranged from 1090–24,542 across biosensors measured).

To perform statistical analysis on these signal parameters, we took the average of the data (e.g. Dynamic Range) across all cells within a given technical replicate. We then pooled the average per technical replicate across all experimental replicates (N ranged from 8–43). A One-Way ANOVA with a Tukey–Kramer Post-Hoc Test for multiple comparisons was used to compare the data. All groups were compared to each other, however only comparisons discussed in the text are presented in the figures. Statistical analysis and P values for all comparisons are available on the REKAR GitHub.

## Results

### Red/far-red-FRET ERK biosensors accurately measure changes in ERK activity

**Fig. 1:**
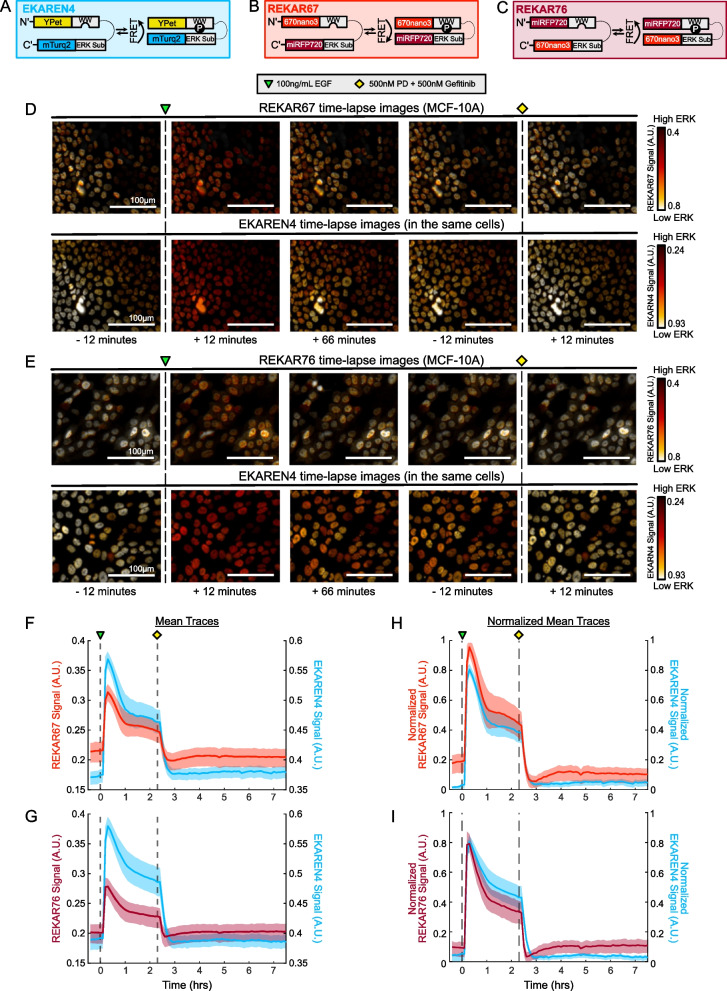
Red/far-red FRET-based ERK biosensor designs and validation. **A**-**C** Graphical representations of the ERK biosensors EKAREN4, REKAR67, and REKAR76. **D **&** E** Pseudo-colored ratiometric images of biosensor activity from cells co-expressing the REKAR and EKAREN4 biosensors. *Top row in each panel* shows REKAR67 or REKAR76 and *bottom row in each panel* shows EKAREN4. ERK activity is indicated by pseudocoloring (white = low FRET, red = high FRET). Images show cells pre-treatment, following EGF stimulation (green triangle), and following MEK/EGFR inhibition (yellow diamond). Times shown are relative to the time of EGF treatment (leftmost 3 columns) or the time of inhibition (rightmost 2 columns). Scale bars are 100 μm. **F**-**G** Average calculated FRET signal of REKAR67 (red) or REKAR76 (maroon) overlaid on the EKAREN4 signal (blue) of the same cells. Treatment with EGF and inhibitors was performed as in **D **&** E**. **H-I** Data from **F **&** G** plotted with each reporter signal normalized for comparison as indicated in Materials and Methods. For **F**—**I**, the bold line represents the mean signal and the lighter shaded regions indicate the 25th −75th interquartile range

To develop FRET-based ERK biosensors that do not overlap with fluorescence in the 400–600 nm range, we constructed REKAR67 and REKAR76 by replacing the Turquoise2 (CFP) and YPet (YFP) fluorophores of EKAREN4 [17] with miRFP670nano3 [28] and miRFP720 [29] (Fig. 1A-C). Because it remains unclear how fluorophore position affects ERK biosensor performance, REKAR67 and REKAR76 were designed with alternate positions to identify the best configuration for imaging properties such as dynamic range (minimum to maximum signal measured). The two constructs were named based on the N or C terminal location of the red fluorescent proteins (N to C terminal order):

1. REKAR67: miRFP670nano3—Phospho-recognition domain (WW)—linker domain—ERK substrate—miRFP720 (Fig. 1B).
2. REKAR76: miRFP720—Phospho-recognition domain (WW)—linker domain—ERK substrate—miRFP670nano3 (Fig. 1C).

We validated REKAR67 and REKAR76 by stably expressing each of these biosensors in MCF-10A cells already expressing the EKAREN4 biosensor and comparing their responses to canonical ERK activators (100 ng/mL EGF) and pathway inhibition (500 nM each of the MEK inhibitor PD-0325901 and the EGFR inhibitor gefitinib) (Fig. 1 D & E; Sup. Videos 1–4). Treatment with EGF caused a sharp increase in EKAREN4, REKAR67, and REKAR76 signals, peaking approximately 30 min after treatment, whereas treatment with PD-0325901 and gefitinib resulted in a decrease to the minimum signal within 20 min (Fig. 1 F & G). To better compare REKAR67 and REKAR76 to EKAREN4, we normalized each ERK biosensor signal (Fig. 1 H&I; see Materials and Methods for details). With this normalization, the rise and fall of both REKAR variants was highly similar to EKAREN4 and to one another. However, a consistent difference between EKAREN4 and both REKAR variants was that, following inhibitor treatment, REKAR levels first fell to a minimum lower than the initial baseline, and then recovered within about 60 min to a level near the baseline. In contrast, EKAREN4 signals fell immediately to the baseline level, without the initial dip observed in REKAR signal. Additionally, we noticed that the 25th-75th interquartile range (IQR) for both REKAR reporters tended to be broader than for EKAREN4 during the pre-stimulation and post-inhibition phases, indicating a greater degree of variation. Nonetheless, despite these minor differences, the similarities of REKAR67 and REKAR76 signals to EKAREN4 signals suggest that both REKAR biosensors have a similar capacity to measure changes in ERK activity.

### REKAR67 and REKAR76 signals are dependent on ERK substrate phosphorylation status

We verified that neither configuration of REKAR acquired unintended off-target phosphorylation sites by generating variants of both sensors in which the phosphoacceptor threonine of the ERK substrate sequence was substituted with alanine. This “T/A” mutation eliminates the ERK phosphorylation target within the biosensors, such that these variants should not exhibit a change in FRET signal when any kinase, including ERK, is active within the cell. We generated cells expressing EKAREN4 and either REKAR67-T/A or REKAR76-T/A (Sup. Fig. 1A & B; Sup. Videos 5–8). Unlike their functional counterparts, neither REKAR-T/A variant responded to EGF or PD-0325901 and gefitinib treatment, while the co-expressed EKAREN4 maintained its responsiveness to both treatments (Sup. Fig. 1C & D). Therefore, the response of both REKAR biosensors is strictly dependent on the phosphorylation of the threonine residue of the ERK substrate sequence. Furthermore, this experiment demonstrated that changes in REKAR sensor signals are spectrally independent from changes occurring in a co-expressed CFP/YFP biosensor.

### REKAR67 and REKAR76 both accurately measure single-cell ERK activity

**Fig. 2:**
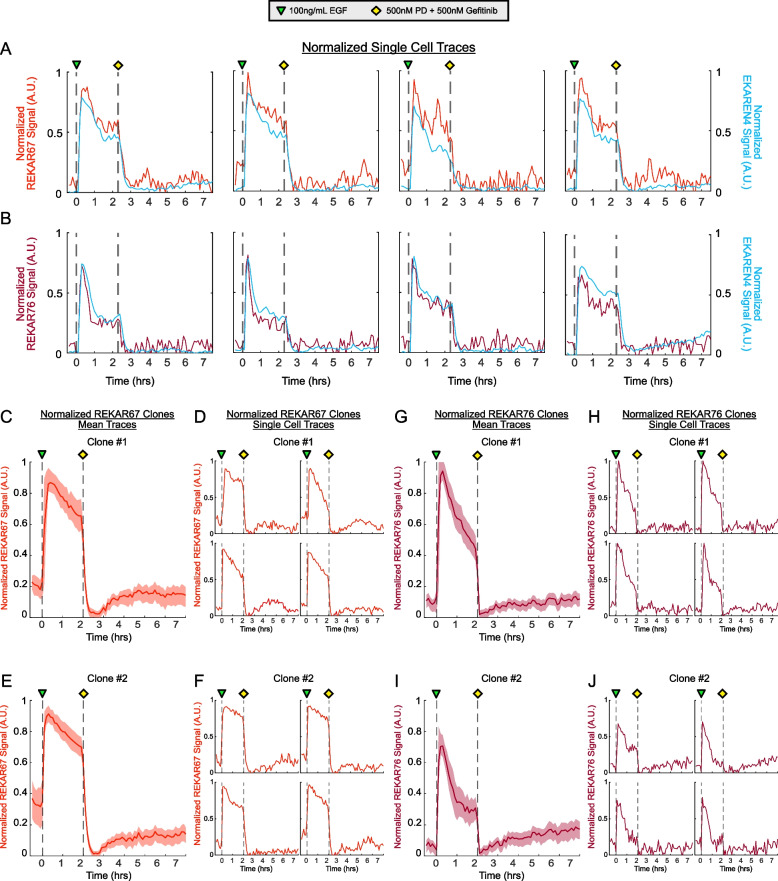
Single-cell REKAR-EKAREN4 and REKAR clone activity traces. **A** REKAR67 and EKAREN4 signals from the same cell. Plots from four representative MCF10A cells expressing both biosensors are shown. Biosensor signals for REKAR67 and EKAREN4 were normalized for comparison (see Materials and Methods) and are overlaid in red and blue, respectively. **B** Four representative single-cell plots from MCF10A REKAR76-EKAREN4 dual-expressing cells, normalized as in **A** and plotted in maroon and blue, respectively. **C**-**F** Normalized miRFP FRET activity of clonal REKAR67 expressing MCF10A cells. **G-J** Normalized miRFP FRET activity of clonal REKAR76 expressing MCF10A cells. For C, E, G, and I, the bold line represents mean signal, and the lighter shaded regions the 25th/75th interquartile range

To further compare the REKAR variants to EKAREN4, we generated visualizations of individual cells, using a similar normalization scheme to the mean plots displayed in Fig. 1 (Fig. 2A&B). In these visualizations, we again found a close concordance between REKAR signals and EKAREN4. Notably, when cells differed from one another in the shape of their EGF-induced ERK activity peak, this shape was shared by both the REKAR and EKAREN4 signals within the same cell. Thus, when used to read out cell-to-cell differences, EKAREN4 and REKAR provide consistent information. However, we also noted that REKAR signals showed a greater degree of high-frequency signal variance at baseline, compared to EKAREN4. This variation in REKAR was not obviously correlated with the EKAREN4 signal in the same cell, suggesting that it may contribute to the broader IQR observed for REKAR reporters, noted in Fig. 1.

To dissect the possible sources of the greater signal variance in REKAR, we isolated clonal populations of REKAR-expressing cells. These clones were less subject to variable expression of the reporter found in the original polyclonal population, which we attribute to expression of the REKAR biosensor from the same genetic locus within each clone. As expected, the average responses of REKAR clones were highly similar to the polyclonal populations from which the clones were derived (Fig. 2C&D). Furthermore, the IQRs for the clones were smaller than for the polyclonal REKAR expressing populations, (Fig. 1F-I). However, it was unclear how the noise characteristics of the clones compared quantitatively to those of EKAREN4, indicating the need for a formal analysis of signal and variance properties.

### Signal-to-noise properties of REKAR67 and REKAR76

**Fig. 3:**
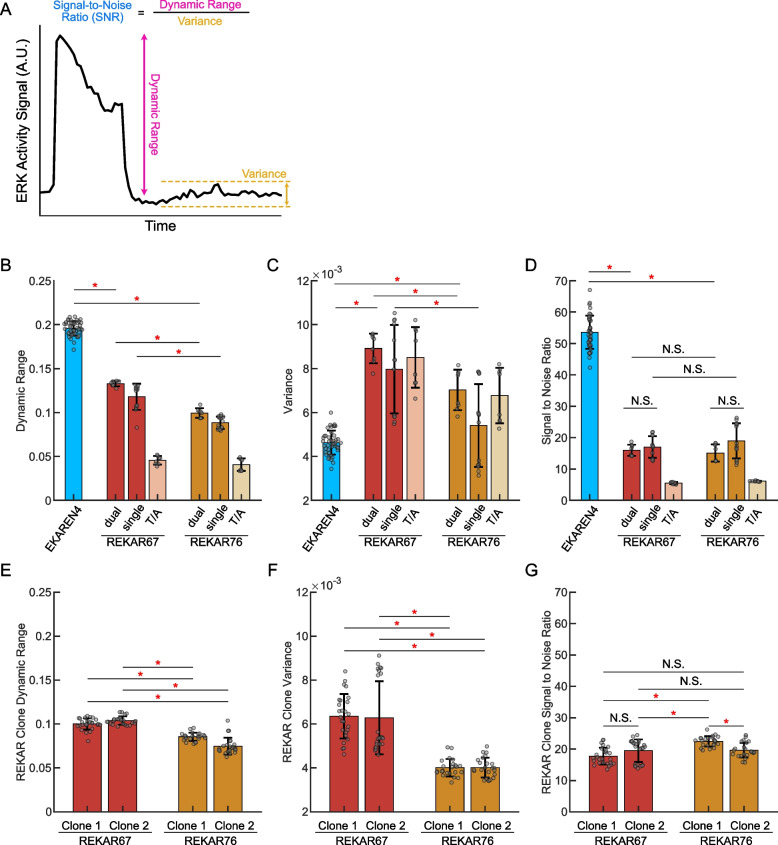
Quantitative analysis of reporter characteristics. **A** Example plot indicating dynamic range (pink) as the distance between maximum and minimum signal, variance (yellow) as calculated using the post-inhibition signal, and *SNR* (blue) as the quotient of the dynamic range and variance. **B-D** Bar plots showing the dynamic range (**B**), variance (**C**), and SNR (**D**) of EKAREN4-, REKAR67-, and REKAR76-expressing cells. All data in **B**-**D** are taken from polyclonal REKAR populations. Columns labeled “dual” indicate measurements of REKAR taken from EKAREN4-REKAR dual biosensor cells, while columns labeled “single” are from cells expressing REKAR only. Columns labeled “T/A” indicate measurements in cells expressing the indicated REKAR T/A mutant variants. EKAREN4 data are from all cell lines expressing EKAREN4, including cells co-expressing biosensors. **E–G** Bar plots showing each REKAR clone’s dynamic range (**E**), signal variance (**F**), and *SNR* (**G**), as indicated. Two clonal REKAR expressing MCF10A cell lines were used per biosensor variant, referred to as Clone 1 and Clone 2 (*N* = 23–26). For all plots**,** red asterisks indicate *P* < 0.05 between the indicated groups as determined by One-Way ANOVA with a Tukey–Kramer Post-Hoc Test for multiple comparisons and gray dots on bars represent the mean of each experimental and technical replicate data (*N* = 8–43)

We next evaluated and compared the *SNR* for EKAREN4, REKAR67 and REKAR76. We defined “Signal” as the dynamic range of the biosensor signal from maximal activation (following EGF stimulation) to maximal suppression of ERK (following a PD and gefitinib treatment). “Noise” was defined as the frame-to-frame variance of the signal following inhibition (that is, the variation at baseline, Fig. 3A), because we expected that true ERK activity would be fully suppressed under these conditions. The observation that the T/A variants exhibited similar Noise values to inhibited cells confirmed that this baseline noise was independent from ERK activity. In polyclonal REKAR populations, we found that REKAR67 had a significantly larger Signal than REKAR76 (0.13 and 0.10 A.U.; *P* < 0.05). Comparatively, EKAREN4 had a larger Signal value of 0.2. As expected, T/A mutants had very low Signal values of < 0.05 (Fig. 3B). REKAR67 also had significantly larger Noise than REKAR76 (0.009 and 0.007 A.U.; *P* < 0.05) (Fig. 3C). From these Signal and Noise values, we then calculated the *SNR* ratio for each biosensor. In these calculations, the higher values of both Signal and Noise for REKAR67 offset each other, such that the two REKAR configurations were comparable in *SNR* (16.0 and 15.1; *P* = 0.998). Furthermore, the differences in REKAR67 and REKAR76 were consistent, regardless of whether the cells were co-expressing EKAREN4 (REKAR single vs. dual; Fig. 3B-D) A similar calculation for EKAREN4 displayed a ~ threefold larger *SNR* than either REKAR variant (EKAREN4 *SNR* = 53.6), due to its greater Signal and lower Noise measurements (Fig. 3D).

Analysis of clonal populations of REKAR-expressing cells resulted in similar conclusions. Both clones of REKAR67 had significantly higher Signal values (0.100, 0.104) than those of REKAR76 (0.085, 0.075) (Fig. 3E). Similarly, REKAR67 clones had significantly higher Noise (0.006, 0.006) than REKAR76 clones (0.004, 0.004) (Fig. 3F). *SNR* values for clones of REKAR67 were 17.7 and 19.5, in comparison to 22.5 and 19.7 for REKAR76 (Fig. 3G). Overall, neither REKAR variant appears to display a consistently greater *SNR* in these clonal populations. Furthermore, the REKAR clones displayed *SNR*s similar to those of the polyclonal population, which were three-fold lower than the *SNR* of EKAREN4. Thus, while clonal expression of REKAR removes some of the variation found in the polyclonal population, a persistent baseline variance is found in all populations of REKAR-expressing cells, indicating a source of noise that we have not yet fully accounted for.

## Discussion

The two red/far-red-FRET biosensors reported here add new capabilities to the existing toolbox of signaling biosensors. We demonstrate that these new constructs perform similarly to existing ERK FRET biosensors in their responses to both stimulus and inhibition and can be easily multiplexed with green- or yellow-range biosensors (e.g., CFP/YFP or GFP-based biosensors). This capability will make it possible to measure ERK activity in tandem with the many cpGFP-based sensors now available for metabolites (including ATP, glucose, lactate, pyruvate, 1,6 bisphosphate [30–34], signaling intermediates and cofactors [35–37], and other kinases [16, 21, 38, 39]. Another red-shifted ERK reporter is also available, termed RAB-EKARev [39, 40]. This biosensor is based on a similar intramolecular binding design, but relies on dimerization-dependent fluorescence of ddRFP, rather than FRET. Overall, RAB-EKARev appears to have similar kinetic responses to growth factor stimulation, but its reversibility following ERK inhibition has not been established, and it was not directly compared to existing ERK biosensors. Additionally, the excitation and emission profile for REKAR67/76 is substantially further red-shifted relative to ddRFP. It should be possible in principle to multiplex CFP/YFP, ddRFP, and miRFP670/720 FRET-based reporters without significant spectral overlap.

While it was previously possible to obtain a readout of ERK activity in the red or far red range using ERK kinase translocation reporters (ERK-KTRs) [41], in which any color FP can be used, this approach requires compromises in the specificity of the reporter and the ability to monitor subcellular activity localization. Like the initial versions of EKAR, ERK-KTRs have some responsiveness to CDK activity [42]. While a modified version may reduce the non-specific CDK1 response in some systems [43], it remains CDK2-responsive in other contexts [44]. The CDK2 responsiveness of ERK-KTR can be corrected mathematically when CDK2 biosensor measurements are also available [42, 45], but this configuration adds substantial complexity to the imaging and data analysis. Moreover, KTRs are not able to provide measurements of kinase activity within specific subcellular regions, as they rely on changes in subcellular localization as their readout mode. Finally, KTR-based reporters can be problematic in situations where cell shapes are irregular or are changing during the experiment, as these changes can affect the ability to reliably sample and segment the cytoplasm without artifacts. Avoiding such artifacts will likely be particularly useful when imaging cells within tissue contexts, whether in vivo or ex vivo. We expect the REKAR biosensors to be particularly useful in these contexts, especially given the greater penetrance of red wavelengths through tissue.

With the limitations overcome by REKAR67 or REKAR76, it should be possible to design many new experiments with paired subcellular measurements for multiple kinases. Interestingly, REKAR67 or REKAR76 also make it possible to monitor ERK activity at different cellular locations simultaneously, because they can be paired with a differentially localized CFP/YFP ERK reporter. For example, ERK activity at the plasma membrane and in the nucleus, which have been reported to be distinct [22, 46, 47], could now be directly correlated within the same cell. While both EKAR and REKAR variants were localized to the nucleus in the dual-reporter experiments shown here, it would be straightforward to add alternate localization motifs to one or both reporters. Like the existing FRET-based phosphorylation sensors, the FRET output of REKAR represents a measurement of the balance between phosphate addition by kinases and phosphate removal by phosphatases. Thus, when interpreting comparative measurements, it will be important to keep in mind that differential FRET signals could result from different phosphatase activities in subcellular locations, as well as from differences in kinase activity [22, 48].

This work raises a number of structural considerations for FRET-based kinase activity sensors. First, the fact that both we and others obtained functional reporters through a simple swap in FPs without the need for substantial optimization suggests that this strategy can be applied more broadly to existing FRET reporters. Additional work will be needed to determine what limits apply to such designs. Optimization of REKAR could prove more difficult than for CFP-YFP biosensors, because miRFPs do not have a simple beta barrel structure permitting circular permutations that can be used to adjust dipole coupling angles. Furthermore, miRFPs rely on the incorporation of an exogenous chromophore (biliverdin), increasing the difficulty of optimizing FP structure to increase quantum yield and thus brightness [49, 50]. The limited quantum yield of miRFPs decreases their relative FRET efficiency, highlighting the need for the development of brighter near-infrared FPs [51]. Regarding biosensor design, the relatively low quantum efficiency of the miRFPs could favor shorter linker domains, which will need to be balanced against the advantage of longer linkers in reducing the coupling in the unphosphorylated state. The smaller size of the miRFP fluorophores could change the way that the biosensor interacts with both kinases and phosphatases, and a more careful examination of how these proportions impact on- and off- rates is warranted. Additionally, the optical properties of miRFP-based FRET reporters will need to be experimentally determined, including the pH sensitivity of the readout, and the fluorescence lifetime characteristics.

Finally, we note the limitation that REKAR sensors appear to be subject to a greater degree of measurement noise. We have not fully pinpointed the source of this noise, but we suspect that it may arise in part from the imaging configuration we used, in which the same excitation wavelength is used to generate both the donor and acceptor images. Relative to the configuration used for the CFP/YFP biosensor, this setup results in an activity calculation that is more subject to spectral cross talk and which may amplify noise. Furthermore, imaging the emission wavelengths for both miRFP fluorophores simultaneously using a dual-camera configuration could reduce noise. Additionally, the miRFP fluorophores require biliverdin as a cofactor. While we found that it is optimal to add exogenous biliverdin, as in the experiments shown here, we also observed sufficient fluorescence without this addition, likely due to trace amounts of biliverdin available in the serum-containing growth medium during cell plating. Because biliverdin availability could change depending on the serum source or washing procedures used, it is an important variable to control to ensure experimental replicability. 

## Supplementary Information

Supplementary Material 1. Figure S1. Non-functional REKAR Control Designs and Validation

Supplementary Material 2. Video 1

Supplementary Material 3. Video 2

Supplementary Material 4. Video 3

Supplementary Material 5. Video 4

Supplementary Material 6. Video 5

Supplementary Material 7. Video 6

Supplementary Material 8. Video 7

Supplementary Material 9. Video 8

## Data Availability

REKAR67, REKAR67-T/A, REKAR76, and REKAR76-T/A plasmids are available from Addgene (plasmid numbers: 223261, 225706, 223262, and 225705, respectively). Cell lines will be provided upon request to the corresponding author (jgalbeck@ucdavis.edu). Image data files collected via NIS elements could not be uploaded due to large file size but are available upon request. All data processing was performed in MATLAB using previously described methods [12, 25, 26]. MATLAB code is available via GitHub repository: https://github.com/Albeck-Lab/REKAR.
