# Supplementary material for: Two novel red-FRET ERK Biosensors in the 670-720 nm range
Source: J Biol Eng. 2025 Nov 13;19:102. doi: 10.1186/s13036-025-00541-9 (PMC12616953; doi:10.1186/s13036-025-00541-9)
Supplement: Supplementary file 1 — Supplementary Material 1. Figure S1. Non-functional REKAR Control Designs and Validation [file 13036_2025_541_MOESM1_ESM.pdf]

Supplemental Figure 1

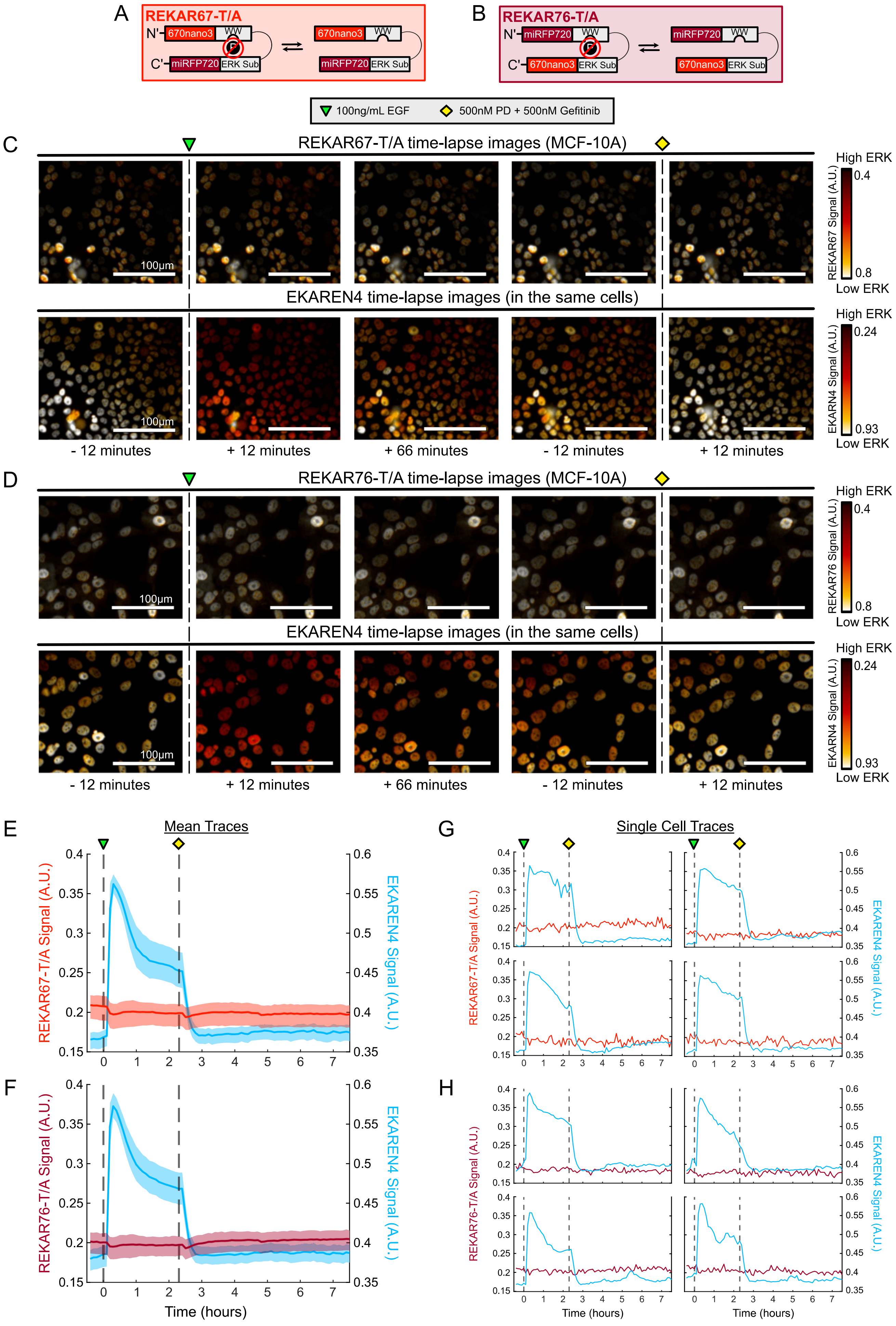

Supplemental Figure 1. Non-functional REKAR Control Designs and Validation. (A-B) Graphical representations of the mutated red-FRET ERK biosensors REKAR67-T/A, and REKAR76-T/A. **(C-D)** Time lapse images of MCF10A cells co-expressing the REKAR-T/A and EKAREN4 biosensors. FRET activity is indicated by pseudocoloring (white = low FRET, red = high FRET). *Top row in each panel* shows REKAR67 or REKAR76 and *bottom row in each panel* shows EKAREN4. Images show cells pre-treatment, following EGF stimulation (green triangle), and following MEK/EGFR inhibition (yellow diamond). Times shown are relative to the time of EGF treatment (leftmost 3 columns) or the time of inhibition (rightmost 2 columns). Scale bars are 100μm. **(E-F)** FRET signal of REKAR67-T/A (red) or REKAR76-T/A (maroon) overlaid on the EKAREN4 signal (blue) of the same cells. The bold line represents mean signal and the accompanying lighter shaded regions are the 25th/75th interquartile range. **(G-H)** Single-cell activity traces from the respective REKAR-T/A-EKAREN4 data presented in **E&F**, overlayed.

SUPPLEMENTAL VIDEO LEGENDS

Supplemental Video 1: MCF-10A cells that simultaneously express both REKAR67 and EKAREN4 ERK biosensors. The movie shows pseudo-colored images made by taking the ratio of miRFP670nano3 and FRET720 channels (REKAR67). Cells are treated with 100 ng/mL EGF 30 minutes into the movie. Cells are also treated with 500 nM PD-0325901 and 500 nM Gefitinib 2.5 hours into the movie. Single frames of this movie were used to make Figure 1 of the paper.

Supplemental Video 2: MCF-10A cells that simultaneously express both REKAR67 and EKAREN4 ERK biosensors. The movie shows pseudo-colored images made by taking the ratio of CFP and YFP channels (EKAREN4). Cells are treated with 100 ng/mL EGF 30 minutes into the movie. Cells are also treated with 500 nM PD-0325901 and 500 nM Gefitinib 2.5 hours into the movie. Single frames of this movie were used to make Figure 1 of the paper.

Supplemental Video 3: MCF-10A cells that simultaneously express both REKAR76 and EKAREN4 ERK biosensors. The movie shows pseudo-colored images made by taking the ratio of miRFP670nano3 and FRET720 channels (REKAR76). Cells are treated with 100 ng/mL EGF 30 minutes into the movie. Cells are also treated with 500 nM PD-0325901 and 500 nM Gefitinib 2.5 hours into the movie. Single frames of this movie were used to make Figure 1 of the paper.

Supplemental Video 4: MCF-10A cells that simultaneously express both REKAR76 and EKAREN4 ERK biosensors. The movie shows pseudo-colored images made by taking the ratio of CFP and YFP channels (EKAREN4). Cells are treated with 100 ng/mL EGF 30 minutes into the movie. Cells are also treated with 500 nM PD-0325901 and 500 nM Gefitinib 2.5 hours into the movie. Single frames of this movie were used to make Figure 1 of the paper.

Supplemental Video 5: MCF-10A cells that simultaneously express both REKAR67-T/A and EKAREN4 ERK biosensors. The movie shows pseudo-colored images made by taking the ratio of miRFP670nano3 and

FRET720 channels (REKAR67-T/A). Cells are treated with 100 ng/mL EGF 30 minutes into the movie. Cells are also treated with 500 nM PD-0325901 and 500 nM Gefitinib 2.5 hours into the movie. Single frames of this movie were used to make Supplementary Figure 1 of the paper.

Supplemental Video 6: MCF-10A cells that simultaneously express both REKAR67-T/A and EKAREN4 ERK biosensors. The movie shows pseudo-colored images made by taking the ratio of CFP and YFP channels (EKAREN4). Cells are treated with 100 ng/mL EGF 30 minutes into the movie. Cells are also treated with 500 nM PD-0325901 and 500 nM Gefitinib 2.5 hours into the movie. Single frames of this movie were used to make Supplementary Figure 1 of the paper.

Supplemental Video 7: MCF-10A cells that simultaneously express both REKAR76-T/A and EKAREN4 ERK biosensors. The movie shows pseudo-colored images made by taking the ratio of miRFP670nano3 and FRET720 channels (REKAR76-T/A). Cells are treated with 100 ng/mL EGF 30 minutes into the movie. Cells are also treated with 500 nM PD-0325901 and 500 nM Gefitinib 2.5 hours into the movie. Single frames of this movie were used to make Supplementary Figure 1 of the paper.

Supplemental Video 8: MCF-10A cells that simultaneously express both REKAR76-T/A and EKAREN4 ERK biosensors. The movie shows pseudo-colored images made by taking the ratio of CFP and YFP channels (EKAREN4). Cells are treated with 100 ng/mL EGF 30 minutes into the movie. Cells are also treated with 500 nM PD-0325901 and 500 nM Gefitinib 2.5 hours into the movie. Single frames of this movie were used to make Supplementary Figure 1 of the paper.
